# Ultrathin monolithic 3D printed optical coherence tomography endoscopy for preclinical and clinical use

**DOI:** 10.1038/s41377-020-00365-w

**Published:** 2020-07-20

**Authors:** Jiawen Li, Simon Thiele, Bryden C. Quirk, Rodney W. Kirk, Johan W. Verjans, Emma Akers, Christina A. Bursill, Stephen J. Nicholls, Alois M. Herkommer, Harald Giessen, Robert A. McLaughlin

**Affiliations:** 1 Australian Research Council Centre of Excellence for Nanoscale BioPhotonics, Adelaide Medical School, University of Adelaide, Adelaide, SA 5005 Australia; 2 Institute for Photonics and Advanced Sensing, The University of Adelaide, Adelaide, SA 5005 Australia; 3 Institute of Applied Optics (ITO) and Research Center SCoPE, University of Stuttgart, 70569 Stuttgart, Germany; 4 South Australian Health and Medical Research Institute (SAHMRI), Adelaide, SA 5000 Australia; 5 Royal Adelaide Hospital, Adelaide, SA 5000 Australia; 6 Monash Cardiovascular Research Centre, Monash University, Melbourne, VIC 3168 Australia; 7 4th Physics Institute and Research Center SCoPE, University of Stuttgart, 70569 Stuttgart, Germany

**Keywords:** Micro-optics, Imaging and sensing, Biophotonics

## Abstract

Preclinical and clinical diagnostics increasingly rely on techniques to visualize internal organs at high resolution via endoscopes. Miniaturized endoscopic probes are necessary for imaging small luminal or delicate organs without causing trauma to tissue. However, current fabrication methods limit the imaging performance of highly miniaturized probes, restricting their widespread application. To overcome this limitation, we developed a novel ultrathin probe fabrication technique that utilizes 3D microprinting to reliably create side-facing freeform micro-optics (<130 µm diameter) on single-mode fibers. Using this technique, we built a fully functional ultrathin aberration-corrected optical coherence tomography probe. This is the smallest freeform 3D imaging probe yet reported, with a diameter of 0.457 mm, including the catheter sheath. We demonstrated image quality and mechanical flexibility by imaging atherosclerotic human and mouse arteries. The ability to provide microstructural information with the smallest optical coherence tomography catheter opens a gateway for novel minimally invasive applications in disease.

## Ultrathin 3D printed endoscope to image tiny spaces in the body

A 3D printed endoscope less than half a millimeter in diameter provides high-resolution images inside narrow arteries without damaging tissue. Miniaturized endoscopes hold great potential for imaging delicate organs, but it is difficult to achieve high-quality imaging with very small components. Jiawen Li at University of Adelaide, Australia, Simon Thiele at University of Stuttgart, Germany, and co-workers used laser 3D microprinting to produce optical components just 125 micrometers in size, including a miniaturized prism that focuses and directs light, and corrects aberrations. This periscope-like design collects 360-degree images by rotating inside a transparent catheter sheath that protects the surrounding tissue. The researchers tested the endoscope by imaging the inside of fresh human and mouse arteries that were severely narrowed by plaque build-up from atherosclerosis, suggesting it could identify risk factors for heart attacks and strokes.

## Introduction

Fiber-optic endoscopes have become an indispensable clinical tool, providing diagnostic images of the internal lumen of hollow organs and real-time guidance during interventions^1–4^. In particular, endoscopes using optical coherence tomography (OCT), which provides depth-resolved imaging, have been used on >410,000 patients to improve clinical outcomes^5^. In addition, OCT has become a widely used tool for assessments in preclinical animal models^6,7^.

Despite these advances, there remains a practical but unmet need for miniaturized high-resolution probes that not only enable the imaging of delicate narrow luminal organs and small animals but also prevent the potentially severe adverse events from trauma arising from endoscope insertion^1,4,7^. Specifically, a high resolution and a large depth of focus are necessary for the effective surveillance of pathological changes^6–8^ but are extremely challenging to achieve with a miniaturized probe^2,8–12^. For example, mouse models are a commonly used animal model for cardiovascular disease^13^, and a miniaturized probe with a 483 µm diameter was reported for intravascular mouse imaging^7^. However, the reported probe was not able to image microstructures deeper than 100 μm owing to its short depth of focus and lacked the resolution to provide a visualization of the relevant structures such as adipose cells^14^, cholesterol crystals (CCs)^15,16^, and connective tissue^7^, which are on the scale of tens of microns.

Current probe fabrication techniques are limited when used for highly miniaturized probes, resulting in spherical aberration, low resolution, or a shallow depth of focus^2,9–12^. In optical design, there is a traditional trade-off of high resolution (large numerical aperture, NA), resulting in a rapidly diverging light beam with a small depth of focus and poor resolution (small NA) used to achieve a large depth of focus. Good designs have been attempted to find the optimal compromise between sufficient resolution and depth of focus^10–12,17^. Unfortunately, with miniaturized probes, the physical aperture of the probe is very small, and no appropriate compromise may exist. The small scale of the optics also makes it very challenging to correct for spherical aberration, which can further degrade the resolution and depth of focus^10^. Usually, the correction of the spherical aberration of a single lens is only possible by using an aspherical lens profile (described by a polynomial instead of a circle equation). This shape, however, is difficult to realize on a fiber tip where lenses are often made using a melting process (e.g., creating a spherical ball lens with a profile described by a circle equation).

In OCT imaging, these issues are exacerbated because endoscopic and intravascular probes are deployed within a transparent catheter sheath, both to protect the animal or patient from trauma as the probe is rotated to perform scanning and to prevent cross-contamination when re-used across multiple animals. Optically, this transparent sheath corresponds to a negative cylindrical lens and causes astigmatism^2,10,18^. Astigmatism increases the decay of the lateral resolution of a miniaturized probe^18^. As a result, the correction of these nonchromatic aberrations is critical to achieve the best possible resolution over the desired depth of focus with a miniaturized probe^9,18^.

Current methods of micro-optic fabrication lack the capability to mitigate these nonchromatic aberrations^10^. Miniaturized OCT probes have been fabricated either by assembling the probe from discrete micro-optical elements (e.g., prisms and lenses)^1,2^ or by splicing fiber lenses, such as gradient-index (GRIN) fiber or fiber-based ball lenses, directly onto the fiber^8,9,19,20^. However, restrictions in the index profile of the GRIN lens/fiber elements^10^ (parabolic profile inherently causing aberrations) and in the shape of ball lenses lead to spherical aberrations and limit the ability to correct astigmatism, resulting in probes that fail to achieve a high resolution over a large depth of focus^10,18^. Ultrafast laser nanostructuring of photopolymers^21–23^ is a viable alternative to create micro-optical components with complex shapes, high accuracy, low surface roughness, and sub-µm precise alignment^24–27^. In earlier work, we showed the potential of this approach to fabricate a discrete optical element that may be glued to an optical fiber to enable OCT imaging^28^. However, this preliminary work did not explore the potential of the technology to correct aberrations, which limit the performance of micro-optics. In addition, the requirement to attach a discrete optical element to the end of an optical fiber presents additional challenges in scaling the manufacture of these probes. The full potential of this technology in the field of endoscopic OCT has not been exploited thus far.

**Fig. 1:**
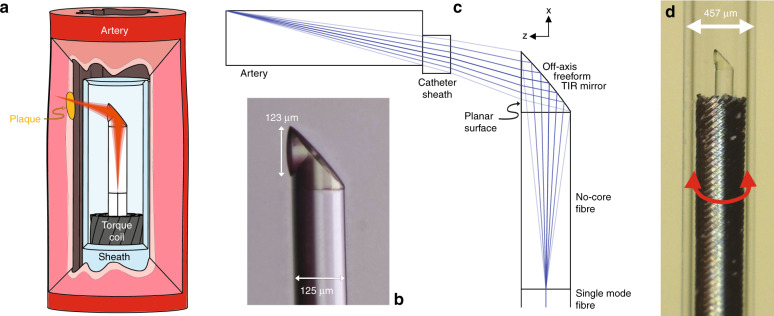
Ultrathin 3D printed endoscope design. **a** Schematic of the 3D printed OCT endoscope inside an artery. **b** Microscope image of the 3D printed off-axis freeform total internal reflection (TIR) mirror on the tip of the no-core fiber that is fusion spliced onto the light-guiding single-mode fiber. **c** Optical design of the system with light exiting the single-mode fiber, expanding in the no-core fiber, being reflected and phase-shaped at the freeform mirror, passing the catheter sheath and focusing into the artery tissue. **d** Photo of the 3D printed OCT endoscope, which rotates and is pulled back to accomplish full 3D OCT scanning

Here, we have developed an ultrathin monolithic OCT endoscope that overcomes the aforementioned limitations by using two-photon polymerization to 3D print 125-μm-diameter micro-optics directly onto the optical fiber (Fig. 1). Freeform micro-optics have been created for correcting the nonchromatic aberrations of highly miniaturized probes, which cannot be fabricated using traditional techniques. The ultrathin OCT endoscope achieved a measured full width at half maximum (FWHM) focal spot size of 12.4 μm and effective depths of focus (the depth range in which FWHM < 2FWHM_min_^29^) of 760 µm (*x* axis) and 1100 µm (*y* axis). The utility of the ultrathin endoscope is demonstrated on both in situ preclinical (mouse) and ex vivo clinical (human) models of cardiovascular disease. We are now able to reveal details of the tissue microarchitecture at depths not previously achieved with such small imaging probes. To the best of our knowledge, this is the smallest aberration-corrected intravascular probe to have been developed.

## Results

### Design and fabrication

A schematic of the ultrathin 3D printed endoscope and a 3D microscope (VHX-1000, Keyence, Japan) image of the 3D printed freeform structure (i.e., without the catheter sheath) are shown in Fig. 1a, b, respectively. A 450 μm length of no-core fiber (Prime Optical Fiber Corporation, China) was spliced onto a 20 cm length of single-mode fiber (SMF28, Thorlabs, USA) to expand the light beam before it reached the 3D printed freeform micro-optic. To achieve splicing of this length of no-core fiber, we first spliced a longer section of no-core fiber to a single-mode fiber and then cleaved it to 450 ± 5 μm using an automated glass processor with an in-line cleaver (Vytran GPX3800, Thorlabs, USA). The beam shaping micro-optic was directly 3D printed onto the distal end of the no-core fiber using a two-photon lithography system (Photonic Professional GT, Nanoscribe, Germany) that was modified with a fiber holder directly attached to the system^30^. The freeform surface of the 3D printed micro-optic redirects and focuses the beam by the total internal reflection (TIR, Fig. 1c). This surface also compensates for the astigmatism caused by the transparent polymer catheter sheath (with an inner diameter of 0.386 mm and an outer diameter of 0.457 mm; produced by Zeus, Inc., USA). The fiber assembly was fixed inside a thin-wall torque coil (with an inner diameter of 0.26 mm and an outer diameter of 0.36 mm, produced by Asahi Intecc Co., Ltd., Japan). The torque coil allows rotational and linear motions to be precisely transferred from the proximal end to the distal end of the imaging probe, thus achieving 3D scanning. The imaging probe rotates freely inside the catheter sheath, which remains stationary and protects the biological tissue during 3D scanning.

### Characterization

**Fig. 2:**
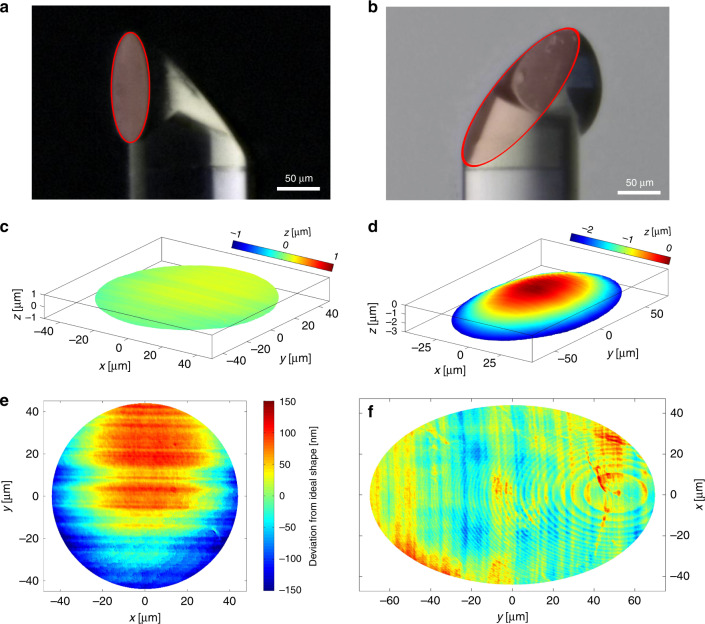
Probe characterization. **a** Microscope image of the probe with the flat front surface highlighted. **b** Microscope image of the probe with the curved TIR surface highlighted. **c** Confocal surface measurement result of the flat interface from **a**. **d** Confocal surface measurement result of a curved TIR mirror surface **b**. **e** Deviation from the ideal shape in the case of the flat surface. The RMS deviation for a beam footprint corresponding to an NA of 0.14 is 43 nm. **f** Deviation from the ideal shape in the case of the TIR surface. The RMS deviation for a beam footprint corresponding to an NA of 0.14 is 19 nm. The visible elliptical ring patterns are caused by the rasterization of the 3D printer within each printing layer and did not have a discernible impact on the optical performance

The surfaces of five replicates were profiled using a noncontact confocal surface profiler (µsurf expert, Nanofocus AG, Germany) after being printed onto fibers with a success ratio of 100%. Representative surface profiles obtained by this profilometer in confocal mode are shown in Fig. 2. The deviation between the design and the printed micro-optic was <34 ± 12 nm root mean square (RMS) in the case of the TIR mirror and <71 ± 52 nm in the case of the planar surface (see Fig. 1) for a beam footprint with NA = 0.14. One possible explanation for the larger surface error of the flat surface is that it is oriented perpendicular to the printing direction, which makes it more sensitive to drifts during printing. Additionally, due to its exposed shape, this surface is more prone to inhomogeneous polymer shrinkage. If these surface errors are translated into a wavefront error ΔW, then the values are close to or below the diffraction limit (ΔW < 0.07λ). This investigation demonstrates the reliable transfer from design to the 3D printed micro-optic and a highly repeatable fabrication performance.

**Fig. 3:**
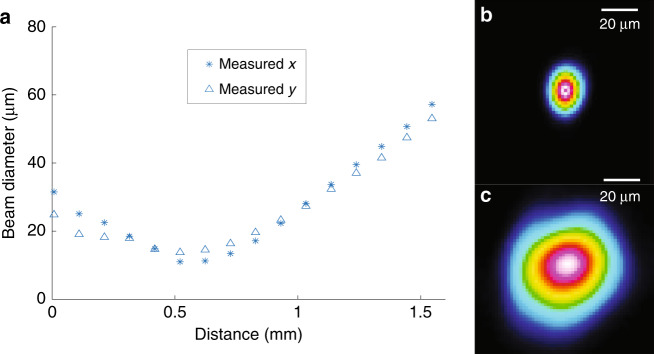
Beam characterization. **a** Beam profiles (FWHM) obtained from the 3D printed OCT probe with the catheter, indicating negligible astigmatism (coordinates shown in Fig. 1). The *x* axis is the distance to the catheter surface. **b** Focal spot profile. **c** Spot profile from a plane ~1.15 mm from where the light exits the catheter surface

The fabricated imaging catheters are intended to be used inside biological samples, where the refractive index of the body fluid is comparable to that of water. Accordingly, the beam profile of the fabricated imaging catheter was measured in water to emulate the conditions inside the body. The imaging catheter was mounted onto custom-designed holder-stage units (Supplementary Fig. 1) to immerse the tip of the imaging catheter in water and ensure that the optical beam from the fiber assembly was incident normal to the CMOS camera (WinCamD-XHR-1310, DataRay Inc., USA). The beam profile measured in water is provided in Fig. 3a. The FWHM beam diameters measured along both directions (*x* and *y* axes, as shown in Fig. 1c) indicate negligible astigmatism. The measured focal spot (with ~12.4 µm FWHM) and working distance (~513 µm) agree well with the simulated values (12.8 µm FWHM and 500 µm working distance). The estimated effective depths of focus calculated by fitting the measured beam diameters with six-degree polynomials (the depth range in which FWHM < 2FWHM_min_^29^) are 760 µm (*x* axis) and 1100 µm (*y* axis).

### Ex vivo imaging of a severely narrowed human artery

**Fig. 4:**
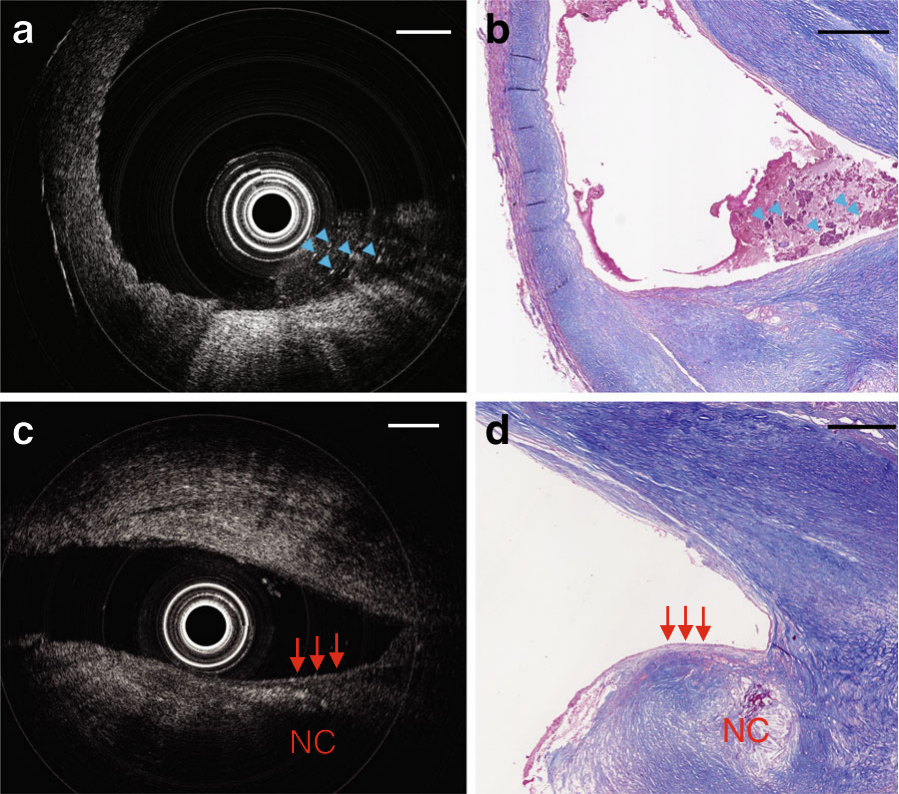
OCT imaging in a severely diseased human carotid artery. **a** Cross-sectional OCT image. **b** Masson’s trichrome staining of adjacent sections shown in **a**; blue arrows indicate thrombus that appears to contain fibrin, platelets, and cellular debris. **c** Another cross-sectional OCT image. **d** Masson’s trichrome staining of the same area shown in **c**; red arrows point to a fibrous cap with an adjacent necrotic core. NC: necrotic core. Scale bar: 500 µm

To evaluate the performance of this ultrathin OCT probe for scanning tissue samples, we imaged a freshly excised human carotid artery. OCT scans of the fresh tissue were compared against histological sections of the fixed tissue. In both this human sample and subsequent mouse scans, histological sections were matched to the OCT scans based on their position relative to the start and end locations of the scan and distance from anatomical landmarks such as the bifurcations and areas of thrombus. The matches were then refined by manually identifying multiple features within each OCT/histology pair. The human sample was obtained from a 75-year-old male undergoing clinically indicated carotid endarterectomy. The study was approved by the Royal Adelaide Hospital Human Research Ethics Committee (R20170715 HREC/17/RA), and informed consent was obtained from the patient. This patient had suffered a stroke 6 months before the endarterectomy procedure. Although this carotid artery revealed severe stenosis, our ultrathin probe was effortlessly delivered through the artery and smoothly rotated and pulled back. In Fig. 4a, an intraluminal thrombus can be visualized, which is in agreement with the corresponding histology photo (Fig. 4b). In Fig. 4c, the diffuse boundary and weak signal region under the high signal region indicate the existence of a necrotic core and a fibrous cap (high signal region). These features are critical for identifying high-risk plaques that may cause heart attacks and strokes^31^. This OCT image reveals a fibroatheroma, which is validated by the corresponding histology (Fig. 4d).

### In situ imaging of healthy and atherosclerotic mouse arteries

**Fig. 5:**
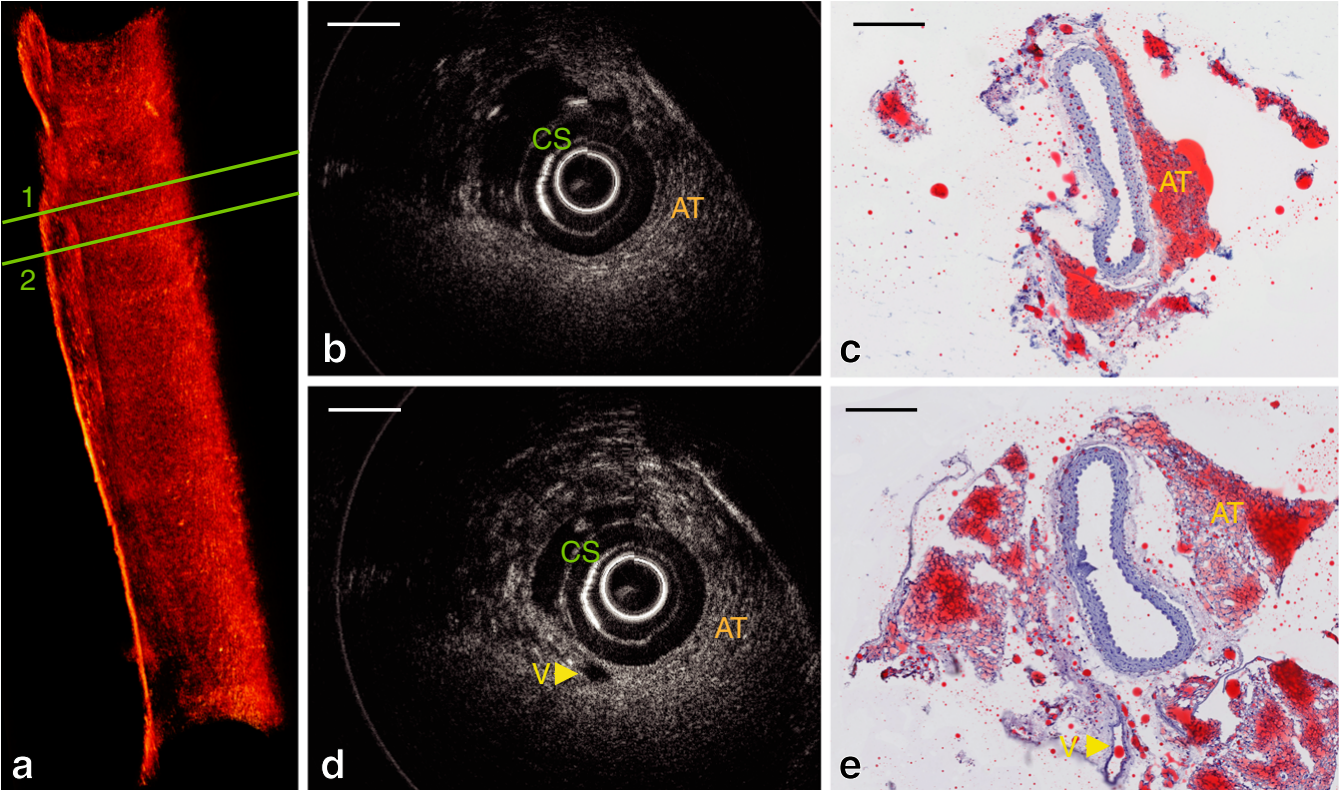
In situ OCT imaging in a normal mouse aorta. **a** Three-dimensional rendering of the volumetric data set acquired with a 3D printed intravascular imaging catheter in a healthy mouse artery with no atherosclerosis. The volume comprises 500 frames of OCT images. A video of this 3D rendering is available as Supplementary Movie 1. **b** Cross-sectional OCT image of region 1 in **a**; **c** corresponding Oil Red O-stained section, where lipid-rich tissue was stained to red color, which indicates the existence of adventitial and perivascular adipose tissue (AT). Tearing in the histology (not OCT) is apparent between the adipose tissue and vessel owing to poor microtome sectioning during histopathology procedures. **d** Cross-sectional OCT image of region 2 in **a**; **e** corresponding Oil Red O-stained sections. AT: adipose tissue that surrounds artery; CS: catheter sheath; V: an additional vessel parallel to the aorta. Scale bar: 250 µm

**Fig. 6:**
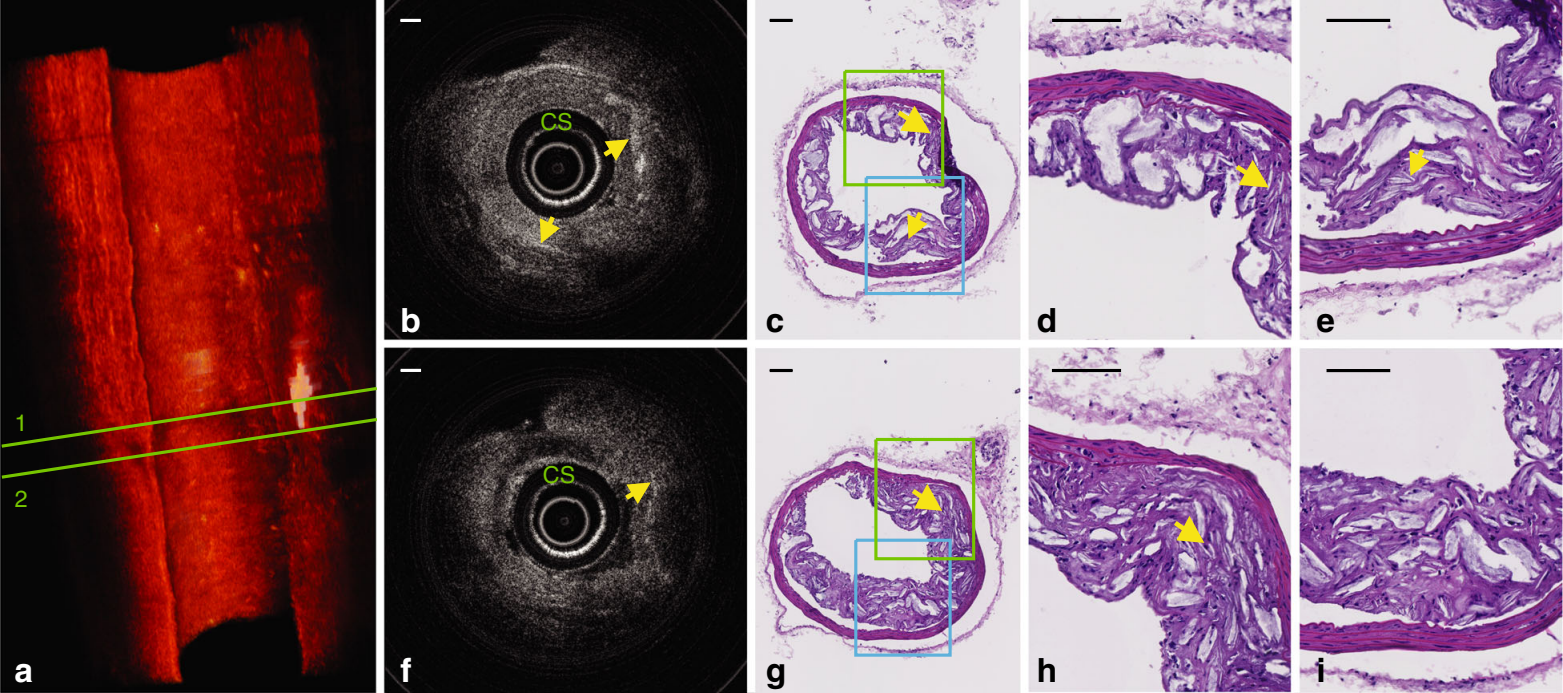
In situ OCT imaging in an atherosclerotic mouse aorta. **a** Three-dimensional rendering of the volumetric data set acquired with a 3D printed intravascular imaging catheter in a diseased mouse artery. The volume comprises 258 frames of OCT images. A video of this 3D rendering is available as Supplementary Movie 2. This rendering reveals distributions of cholesterol crystals, which are indicated by white. **b** Cross-sectional OCT image of region 1 in **a**; **c** corresponding hematoxylin and eosin (H&E) histology image; **d** zoomed green region in **c**; **e** zoomed blue region in **c**. **f** Cross-sectional OCT image of region 2 in **a**; **g** corresponding H&E histology image; **h** zoomed green dashed region in **g**; **i** zoomed blue dashed region in **g**. Yellow arrows point to cholesterol crystals; CS: catheter sheath. Scale bar: 100 µm

We also conducted OCT imaging of mouse thoracic aortas in situ, preserving the in vivo anatomical configuration to emulate in vivo conditions. Animal tissues were obtained from animals killed as part of a different research project approved by the South Australian Health and Medical Research Institute Animal Ethics Committee (SAM188), and tissues were utilized under institutional tissue sharing protocols. To avoid high optical scattering by red blood cells, the blood in the arterial tree was depleted by saline flushing before the artery was scanned. Thanks to the ultrathin footprint of the 3D printed OCT probe, imaging without any obvious rotational distortion (see 3D reconstructions in Figs. 5 and 6) was successfully performed in these very small arteries. The thinnest inner diameter of these arteries was ~0.5 mm (as shown in Fig. 6c, g). After imaging, the arteries were dissected, embedded in mounting compound, cut into 5 μm sections, and stained using hematoxylin and eosin and oil red O (lipid/adipose stain) for histological analysis. These sections were compared with the corresponding OCT images.

Figure 5a shows a 3D volume rendering of the OCT pullback. Voxel transparency and color are specified as a function of the OCT intensity. Representative cross-sectional OCT images obtained in a healthy mouse artery with no atherosclerosis internally within the vessel are shown in Fig. 5b (obtained at Region 1 in Fig. 5a) and Fig. 5d (obtained at Region 2 in Fig. 5a), highlighting microstructural features of and around the artery, including adipose tissues. Figure 5b and d show thick densely packed adipocytes (“AT”), which have a typical diameter of 15–25 μm, appearing as a fine honeycombed texture in the OCT, indicating that this 3D printed probe could identify these microstructural features in situ. These features agree with the histological analysis (Fig. 5c, e). This result is a proof-of-concept demonstration that adipocytes in and beyond adventitia can be imaged by OCT. This is significant because adventitial and perivascular adipocytes have been found to affect the development of atherosclerosis^32,33^.

Example cross-sectional OCT images obtained in an atherosclerotic mouse artery are shown in Fig. 6b (obtained at Region 1 in Fig. 6a) and Fig. 6f (obtained at Region 2 in Fig. 6a), revealing microstructural features inside the plaques, including CCs. CCs are abundant in high-risk plaques that are unstable and likely to rupture and cause a heart attack^34^. CCs were identified as thin, linear structures with high OCT backscattering without attenuation within the plaque^16^. Shallow CCs were observed at the 2 o’clock position of Region 1 (top yellow arrow in Fig. 6b). CCs deep inside plaques were observed at the 7 o’clock position of Region 1 (bottom yellow arrow in Fig. 6b) and the 2 o’clock position of Region 2 (top yellow arrow in Fig. 6f). The locations of CCs were also validated by corresponding histological analysis (Fig. 6c–e and Fig. 6g–i). The findings confirm that biologically relevant microstructures, such as adipocytes (Fig. 5) and CC (Fig. 6), are still distinguishable up to 500 μm deep into the tissue.

## Discussion

In this work, we developed a monolithic, ultrathin high-resolution endoscopic OCT probe by directly writing freeform micro-optics onto a single-mode fiber terminated with no-core fiber. Alternative approaches have recently been used to produce endoscopic probes with a metalens or a freeform micro-optic^10,28^, but both suffer limitations. The outer diameter of the metalens design^10^ is >20 times larger, which is too large to safely scan inside small and delicate ducts and vessels of interest. In contrast, the freeform OCT micro-optic that we reported earlier^28^ did not address the challenge of correcting the astigmatism generated by a catheter sheath or realizing diffraction-limited focusing (Supplementary Fig. 2). In addition, these previously demonstrated nanofabricated optics^10,28^ were designed as discrete elements that must be manually aligned and assembled with fibers by optical adhesives, a process that may take a long assembly time and suffer from poor reproducibility^1^.

Our technique avoids manual alignment between the fiber and micro-optic and ensures sub-micron alignment precision^35^. This technique is the first practical and reliable fabrication method for aberration-corrected highly miniaturized endoscopic probes. The reliability and versatility of our technique leads to a plethora of functionalities that are not feasible with conventional probe fabrication methods (e.g., correction of nonchromatic aberration, beam shaping, and color separation via diffractive elements). One such example, as demonstrated in this work, is to enable the correction of spherical aberration and astigmatism, which ensures high-resolution imaging over a long depth of focus (beam diameters >30 μm for over 1000 μm). We demonstrated the usefulness of the probe in mouse thoracic arteries where biologically relevant microstructures, including adipose cells and CCs, were visualized up to 500 μm deep into the tissue for the first time. Previously, intravascular OCT probes used in mice had a penetration depth of ~250 μm but were only able to visualize biologically relevant microstructures to a depth of ~100 μm^7^. We note that the image penetration depth in the mouse sample (Fig. 5) was less than that in the human tissue (Fig. 4). We hypothesize that this may be owing to the high density of small adipose cells in the mouse tissue resulting in many optical interfaces at the boundary of each lipid-filled cell, which cause higher optical scattering and attenuation.

Apart from the value of using imaging probes in small animals, this ultrathin aberration-corrected probe can also allow safe access to delicate but difficult-to-reach organs, enable a high-resolution cross-sectional imaging capability, and potentially lead to enhanced patient safety and improved health outcomes. The ultrathin high-resolution intravascular imaging of plaques and aneurysms will provide critical microstructural information for diagnosis and guiding treatments^1,2,15,36–39^. In light of the increase in neurointerventions, an imaging catheter with a diameter of >0.6 mm is likely to provide safe access to the internal carotid artery and its branches^37^, where 85% of intracranial aneurysms arise^40^. Such small probes may also offer better access in severely stenotic humans. Moreover, pulmonary applications, including guiding biopsy in peripheral nodules^41^ and imaging inside of the cochlea in the ear^42^, are now within reach.

The ultrathin probe demonstrated in this work was designed to achieve anastigmatic performance in a 1310 nm (center wavelength) intravascular OCT device. Beyond this example, this versatile fabrication method can be used to fabricate endoscopic probes for other optical imaging systems, such as multiphoton imaging and ultrahigh-resolution OCT. Most multiphoton endoscopes require aspherical freeform lenses^43,44^. Reaching small lumens and delicate organs, however, is hampered by current lens fabrication techniques;^43^ these techniques are unable to make sufficiently compact (<1 mm) aspherical freeform lenses to compensate for off-axis aberrations^44^. This 3D printing technology for aberration-corrected imaging endoscopes may make it possible to realize multiphoton imaging inside small luminal organs, allowing for subcellular resolution and molecular specificity deep inside the body. Furthermore, there is a compelling need for extending the depth of focus in ultrahigh-resolution endoscopic imaging probes. For example, ultrahigh-resolution OCT (also referred to as micro-OCT, typically built with a supercontinuum laser centered at ~800 nm) has recently been the subject of intense research in clinical fields, including oncology, cardiology, and otology^8,11,12,15,17,42,45,46^. However, the fabrication of micro-OCT probes, especially in a highly miniaturized format, remains challenging^11,17,42^. The fabrication of unconventional optical elements (e.g., axicon)^45,46^ or the phase/amplitude modification of the imaging system’s pupil^11^ are difficult to reliably manufacture by conventional lens fabrication methods^12^.

We believe that the monolithic freeform 3D microprinting technique presented in this paper has the potential to fulfill this unmet need and accelerate the clinical translation of OCT technology. Beyond this, additional features such as antireflective coatings may be integrated through the fabrication of periodic sub-wavelength structures on the surface of the freeform optics^47^. These capabilities are further expanded by recent work to integrate multiple photoresists into the microprinting process, allowing highly localized customization of the optical properties^48^. Fully automated manufacturing of such probes is feasible using published methods that detect the precise location of the core and outside rim of optical fibers and enable the automated positioning of the 3D printed optics directly onto the cleaved fiber ends^49^.

## Materials and methods

### Design and simulation

The freeform surface was designed and optimized within the optical design software ZEMAX (version 13, Zemax LLC, USA). Although the initial design was optimized in sequential mode, ghost reflections and stray light were evaluated in a subsequent step in non-sequential mode. To function as a TIR surface, the freeform surface is tilted such that all incoming rays fulfill the condition for TIR. More specifically, the angle of the incoming rays relative to the local surface normal needed to be greater than the critical angle of the photoresist surface toward air (41.8°). The surface was tilted by 50° to include some error budget and ensure that surface imperfections do not lead to a local violation of the TIR condition. In addition, the astigmatism caused by the catheter sheath is compensated for by breaking the rotational symmetry of the focusing TIR surface (i.e., the surface can have a shorter focal length for rays within the YZ plane than for rays within the XZ plane, see the coordinate system in Fig. 1). The shorter focal length fits the focal length extension coming from the negative cylindrical lens, which is the catheter sheath, and leads to the compensation of astigmatism. An XY-polynomial of the following form was used as a parametric description of the surface z:

1 $$\begin{array}{l}z = \frac{{c(x^2 + y^2)}}{{1 + \sqrt {1 - c^2(x^2 + y^2)} }}\\ + a_{20}x^2 + a_{02}y^2 + a_{21}x^2y + a_{03}y^3 + a_{40}x^4\end{array}$$

Optimization of the spot size resulted in the parameters shown in Table 1.

**Table 1:** Optimized surface parameters of the 3D printed optic specified in Eq. 1

c	−1.2558 mm^−1^
a_20_	−0.5558 mm^−1^
a_02_	0.1785 mm^−1^
a_21_	0.1754 mm^−2^
a_03_	0.135 mm^−2^
a_40_	−0.83 mm^−3^

The air interface following the TIR mirror was chosen to be flat to reduce complexity. As the light beam passes this interface under an angle of ~10°, the reflected light will not be refocused into the single-mode fiber core, and ghost artefacts are avoided. The same holds true for the subsequent interfaces of the catheter sheath.

For OCT imaging, wavelengths within the range of 1.25–1.35 µm were used. Focusing is diffraction limited with a designed RMS wavefront error of <0.002λ and an effective NA in water of 0.07. The design was optimized for a single-mode fiber NA of 0.2, which is well above the data sheet NA of the SMF28 fiber of 0.14 and ensures that most of the Gaussian beam is guided into focus.

### Fabrication

The resulting design was exported into a computer aided design file format and further refined with the software Solidworks (version 2017, Dassault Systèmes, France). After mounting the fiber in the Nanoscribe system and manually identifying the bounds of the cleaved facet, the print was aligned by determining the center of the no-core fiber. Optical alignment is carried out by illuminating the opposite end of the fiber and observing the end facet with a CCD camera, as described in our earlier work^30^. For the highest surface quality, the printing speed was reduced, and one print required ~90 min to complete. The printing parameters were selected based on earlier work on optimizing the fabrication with a range of lens geometries^50^. There is no additional curing time. After printing, the fiber is simply rinsed in developer to remove unexposed resist. The surfaces were not polished or treated after printing, as the two-photon polymerization was found to give optical quality surfaces without additional post-processing^30,51^. The optical loss of the 3D printed material (loss per unit length and extinction coefficient) has been characterized previously^28,52^. After fabrication, the optics and fiber are enclosed within a plastic catheter sheath, which minimizes any mechanical forces on the optics, especially during imaging. We did not observe any issues with the mechanical stability of the 3D printed optics during imaging experiments and note that it takes a significant mechanical force to detach the 3D printed optics from the fiber. The total fabrication time, including the setup of the fiber, printing and rinsing, was ~120 min.

The lateral voxel size of a print is in the range of 50–200 nm and will depend upon the microscope objective, the photoresist, the laser wavelength and the laser power. The vertical voxel size depends on the NA of the writing objective of the Nanoscribe printer. For our setup, the vertical voxel size is typically 2.7× larger than the horizontal voxel size.

More information regarding the direct fabrication process on fibers can be found in ref. ^30,51^.

### OCT system

The light source, spectrometer, data acquisition card, and workstation of a commercially available OCT scanner (Telesto III, Thorlabs GmbH, Germany) were used for imaging. The spectral bandwidth of our OCT system is 136 nm (with a central wavelength of 1300 nm), and the manufacturer-specified axial resolution of the OCT system is 7.3 μm in air (with Hann Window to perform spectral shaping). The axial resolution is limited by the optical coherence tomography system (predominately the light source and spectrometer) and not by any limitation in the spectral bandwidth arising from the use of the 3D printed micro-optics. This imaging system was interfaced to a custom-built reference arm with an optical pathlength that matched the 3D printed probe sample arm. The power of the OCT beam into the sample arm was 5.8 mW. A sensitivity of 98.4 dB was achieved, with details provided by Supplementary Figs. 3 and 4. The dynamic range of the OCT images was measured to be 52 dB. Volumetric OCT data were acquired using custom software implemented in the C++ language, with the OCT data acquisition synchronized by trigger pulses from a 2D motion (rotation and pullback) control unit. The unit comprised a counter rotation motor^14^ and a linear stage to rotate and pullback the 3D printed probe. The imaging speed of the ex vivo and in situ experiments was one frame per second, which was limited by the speed of the counter rotation stage.

## Supplementary information

Supplementary information

Supplementary movie 1

Supplementary movie 2

## Data Availability

All data needed to evaluate the conclusions in the paper are present in the paper. Additional data related to this paper may be requested from the authors.
