# Supplementary material for: Ultrathin monolithic 3D printed optical coherence tomography endoscopy for preclinical and clinical use
Source: Light Sci Appl. 2020 Jul 20;9:124. doi: 10.1038/s41377-020-00365-w (PMC7371638; doi:10.1038/s41377-020-00365-w)
Supplement: Supplementary file 1 — Supplementary information [file 41377_2020_365_MOESM1_ESM.docx]

**Supplementary Information for**

**Ultrathin monolithic 3D printed optical coherence tomography endoscopy for preclinical and clinical use**

Jiawen Li^1,2*^, Simon Thiele^3^, Bryden C. Quirk^1,2^, Rodney W. Kirk^1,2^, Johan W. Verjans^1,4,5^, Emma Akers^4^, Christina Bursill^1,4^, Stephen J. Nicholls^6^, Alois M. Herkommer^3^,

Harald Giessen^7^, Robert A. McLaughlin^1,2^

**Affiliations**

^1^Australian Research Council Centre of Excellence for Nanoscale BioPhotonics, Adelaide Medical School, University of Adelaide, Adelaide, SA, 5005, Australia

^2^Institute for Photonics and Advanced Sensing, The University of Adelaide, Adelaide, SA, 5005, Australia

^3^Institute of Applied Optics (ITO) and Research Center SCoPE, University of Stuttgart, 70569 Stuttgart, Germany.

^4^South Australian Health and Medical Research Institute (SAHMRI), Adelaide, SA, 5000, Australia

^5^Royal Adelaide Hospital, Adelaide, SA, 5000, Australia

^6^Monash Cardiovascular Research Centre, Monash University, Melbourne, VIC, 3168, Australia

^7^4th Physics Institute and Research Center SCoPE, University of Stuttgart, 70569 Stuttgart, Germany

* Correspondence: jiawen.li01@adelaide.edu.au

**File name:** Supplementary Information Description.

**File name:** Supplementary Movie 1 Description: 3D reconstruction movie of an atherosclerotic mouse aorta as described in Figure 5.

**File name:** Supplementary Movie 2 Description: 3D reconstruction movie of an atherosclerotic mouse aorta as described in Figure 6.

**1. Experimental setup for beam profiling in water**


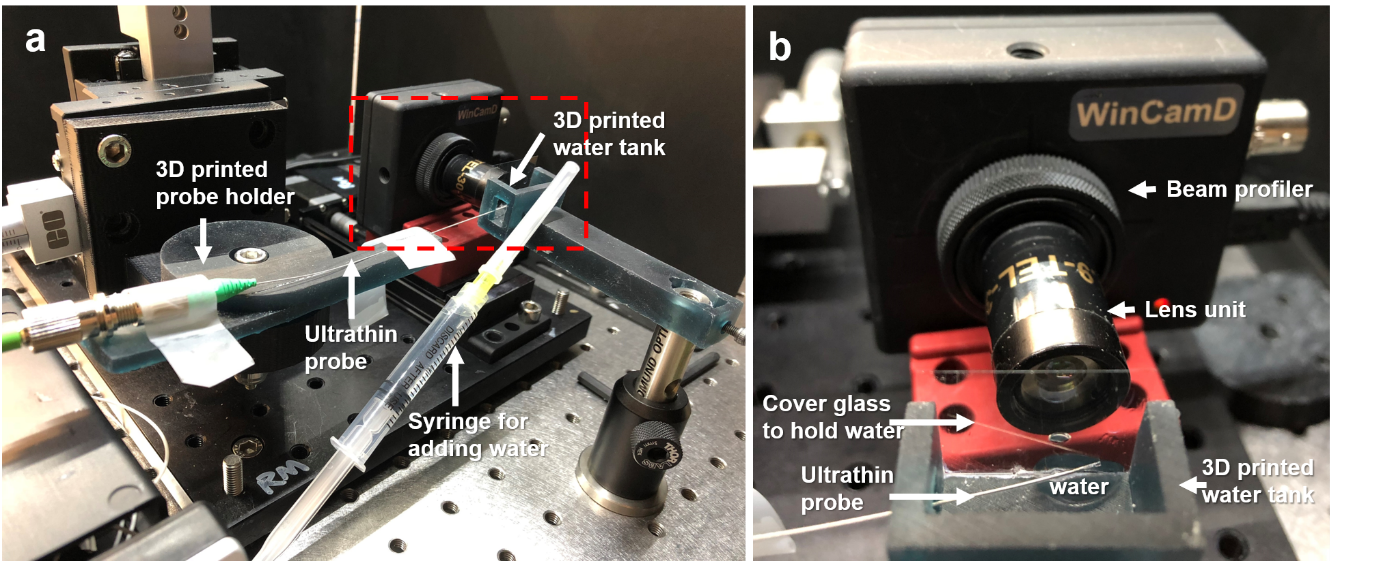


**Supplementary Figure 1.** **Experimental setup for beam profiling in water.** **a** Entire setup with probe. **b** Zoomed red dashed box region in a. During beam profiling, the ultrathin probe was mounted onto a 3D printed probe holder to ensure that the optical beam from the probe was incident normal to the lens unit (LP2-1:1.9-TEL-30-16, DataRay Inc., USA) and the CMOS chip of the beam profiler (WinCamD-XHR-1310, DataRay Inc., USA). The beam profiler camera has a pixel size of 3.2 μm x 3.2 μm and a lens-unit magnification of 1.9x. The beam profiler was moved axially by a linear translation stage (M2DU-50, DataRay Inc., USA) to step through the beam waist.

**2. Comparing the beam profiles of this monolithic aberration-corrected probe with our previous work Ref 28**

**
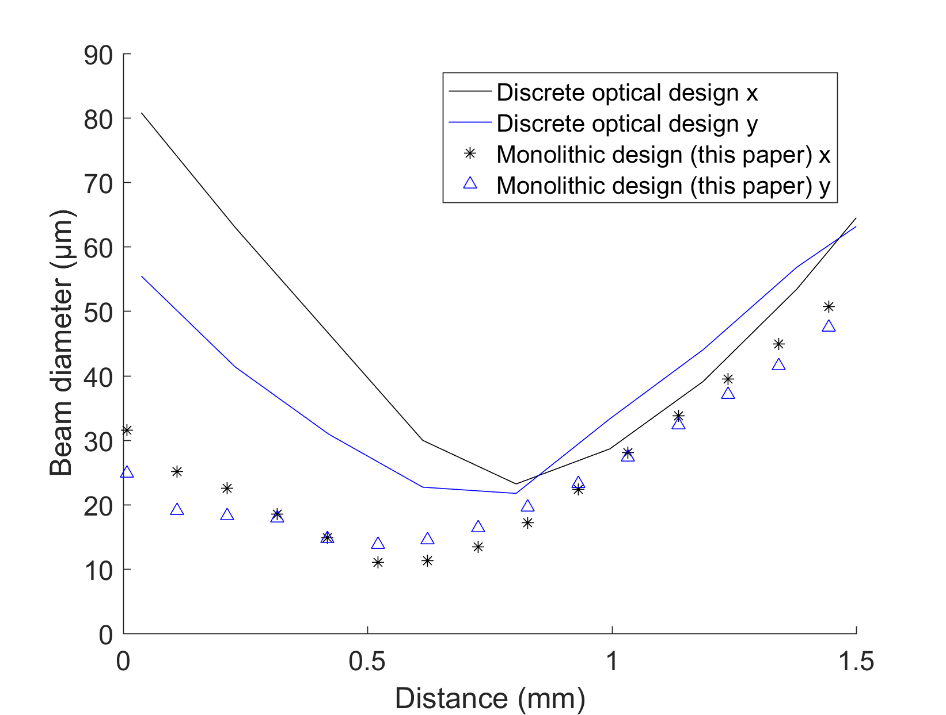
**

**Supplementary Figure 2. Beam profiles of this monolithic aberration-corrected probe and of our previous work** (Ref 28 in the main text). The lateral resolution of this aberration-corrected probe is 12 µm, compared to 23 µm in Ref 28. In addition, astigmatism is much more obvious with Ref 28 design. Note that the size of micro-optics in the Ref 28 is almost 300 µm, more than twice of that presented in this work.

**3. Sensitivity measurement of the OCT system combined with the 3D printed probe**

The sensitivity of the system when using the probe is measured to be at least 98.4 dB.

The sensitivity was determined for the water-immersed intravascular probe by measuring the signal-to-noise ratio (SNR) of a reflection from an interface of fused-silica (n=1.447) and water, resulting in a −27.5 dB Fresnel reflection (see **Supplementary Figure 3** for the setup). During measurement, a double-pass attenuation of -25.9 dB was applied to avoid this reflection from silica-water interface to saturate the OCT spectrometer.


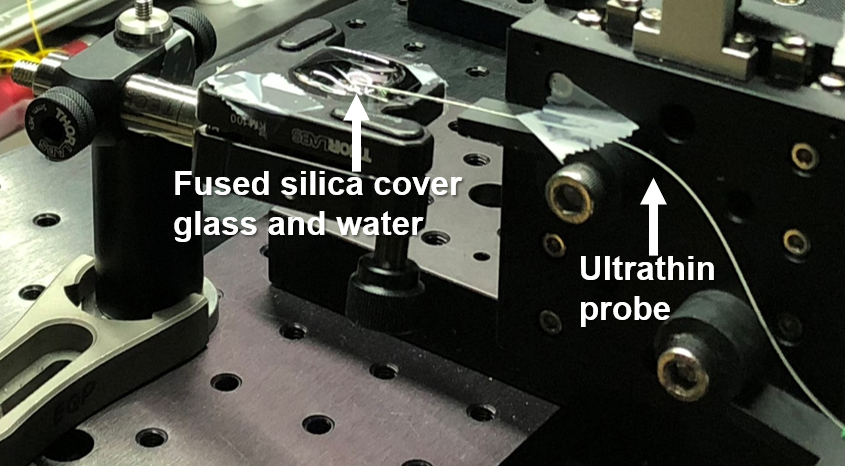


**Supplementary Figure 3. Setup for sensitivity measurement**

The solid line below shows the averaged A-scan signal obtained when the water/silica interface (Peak 1) was at a distance of approximately 500 μm in water from the catheter sheath (Peak 0). Note that the Peak 2 was generated by the back interface of the fused-silica sample (silica/air interface). The SNR at Peak 1 was approximately 45 dB. The sensitivity of the combined OCT system and 3D printed probe was calculated to be 98.4 dB.


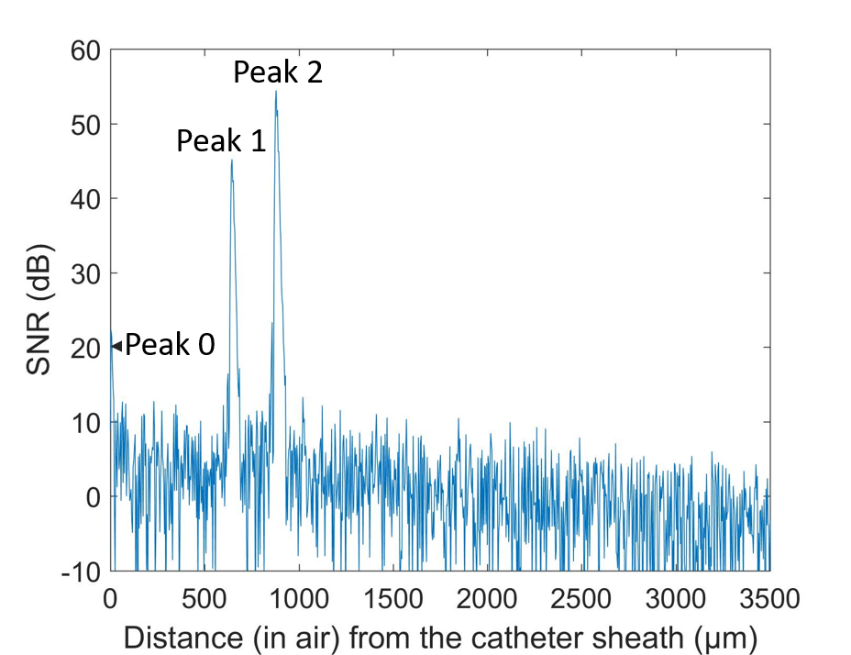


**Supplementary Figure 4. SNR of the water-silica interface** measured by the ultrathin 3D printed probe being immersed in water and a double-pass attenuation of -25.9 dB being applied
